# The theory, direction, and magnitude of ecosystem fire probability as constrained by precipitation and temperature

**DOI:** 10.1371/journal.pone.0180956

**Published:** 2017-07-13

**Authors:** Richard Guyette, Michael C. Stambaugh, Daniel Dey, Rose Marie Muzika

**Affiliations:** 1 School of Natural Resources, University of Missouri, Columbia, Missouri, United States of America; 2 Northern Research Station, United States Forest Service, Columbia, Missouri, United States of America; Pacific Northwest National Laboratory, UNITED STATES

## Abstract

The effects of climate on wildland fire confronts society across a range of different ecosystems. Water and temperature affect the combustion dynamics, irrespective of whether those are associated with carbon fueled motors or ecosystems, but through different chemical, physical, and biological processes. We use an ecosystem combustion equation developed with the physical chemistry of atmospheric variables to estimate and simulate fire probability and mean fire interval (MFI). The calibration of ecosystem fire probability with basic combustion chemistry and physics offers a quantitative method to address wildland fire in addition to the well-studied forcing factors such as topography, ignition, and vegetation. We develop a graphic analysis tool for estimating climate forced fire probability with temperature and precipitation based on an empirical assessment of combustion theory and fire prediction in ecosystems. Climate-affected fire probability for any period, past or future, is estimated with given temperature and precipitation. A graphic analyses of wildland fire dynamics driven by climate supports a dialectic in hydrologic processes that affect ecosystem combustion: 1) the water needed by plants to produce carbon bonds (fuel) and 2) the inhibition of successful reactant collisions by water molecules (humidity and fuel moisture). These two postulates enable a classification scheme for ecosystems into three or more climate categories using their position relative to change points defined by precipitation in combustion dynamics equations. Three classifications of combustion dynamics in ecosystems fire probability include: 1) *precipitation insensitive*, 2) *precipitation unstable*, and 3) *precipitation sensitive*. All three classifications interact in different ways with variable levels of temperature.

## Introduction

The relative role of climate in understanding wildfire is often presented in terms of vegetation, history, ecology, policy and topography [1–5]. These studies and many others have great value, but often do little to address the primary aspect of wildland fire, i.e. that as a physical -chemical reaction, the physics and chemistry of the fire process affects combustion dynamics. Indeed, separating the primary nature of combustion reactions and climate is difficult and therefore the close coupling is often left unacknowledged or misunderstood. In addition, using the many accepted standards of fire interval analyses in fire history studies has yielded much important theory [6–9]. However, when the data provided through fire history reconstruction are considered as frequency data in a physical chemistry context it opens up the well-studied world of classic reaction chemistry with new ecosystem metrics and relevant process equations. The work describes here uses the concepts physical chemistry as a foundation for modeling combustion processes in ecosystems. The effort presented here transforms ecosystem metrics and theory from fire history data. Transcending fundamental fire ecology to more quantitative ecosystem combustion dynamics that reflect the base conditions of fire reactions creates potential approaches to understand ecosystem level climate–fire interactions. This detailed perspective on the physical chemistry of ecosystem wildfire brings with it quantitative intuitive and non-intuitive interpretations of common assumptions about wildland fire.

The primary objectives of this study are to: 1) develop and illustrate temperature/precipitation interactions based on physical and combustion chemistry, 2) present the relevance and value of hybrid process and empirical modeling using basic chemistry and statistics and, 3) state and discuss innovative ecosystem combustion theory gained though modeling and observations. We analyze graphical representations of interdependent phenomena in combustion dynamics to illustrate the strengths of graphic analysis to highlight forcing between precipitation, temperature and wildland fire using an empirically developed simulation graph to explore a partial ecosystem fire process.

## Methods and materials

### Background equations

A review of the general and most advanced version of the three variable Physical Chemistry Fire Frequency Model (PC2FM) method will aid in its use in developing (Eq 1) fire probability [10–12] in a given climate with 170 fire history sites for empirical validation. The formulation, calibration and validation of the PC2FM began by decomposing wildland fire into two components, a reaction environment (temperature and precipitation) and an estimate of reactant concentration (fuel and oxygen). The PC2FM uses the Arrhenius equation $\left(k=A_{0}exp^{-Ea/RT}\right)$ as the concept for calculating the effects of physical- chemistry on the reaction environment [11]. This component of the PC2FM we call the *ARterm* (Eq 1). The second PC2FM component, *PT*_*rc3*,_ represents an estimate of reactant availability (concentration, moisture, oxygen) based on reaction-concentration rate equation (rate = [A][B]). The probability version of PC2FM equation used here is expressed as:

$$AFrP\;=\;1/(-4.3\;\;+\;\;\left(1.7\;e\;-\;28\;x\;ARterm\right)\;\;+\;\;(92\;x\;\left(0_{\text{2}}\;\left(1/P^{\text{2}}/T\right)\right)$$ (1)

where: *AFrP* = annual fire probability in a 1.2 km^2^ area, *ARterm* represents the reaction environment as: A_o_ = (*P*^2^) / (*pp*O_2_) partial pressure of oxygen in kPa, as estimated by elevation, A_o_e^(Ea/(R x *T*)^, e = 2.718, E_a_ = 132 kJ (activation energy), R = 0.00831 kJ mol^-1^K^-1^ (Universal Gas Constant), T = average annual monthly mean daily maximum temperature in K, *P* = annual precipitation in cm; *PT*_*rc3*_ represents the estimated reactant concentration and quality: [(*pp*O_2_)^2^] x [1 / (*P* x *T)*]. Although this equation predict *AfrP* or annual fire probability, it may be expressed as mean fire interval (MFI), the average number of years between fires. MFI = 1/*AFrP*. These are not, however, surrogates for fire intensity or severity. This model simply expresses the likelihood of fire, not the consequences.

Regression coefficients provide calibrated ‘bridges’ between the conceptual chemistry and the effects of climate on ecosystems. Multicollinearity among predictor variables was negligible, the variance inflation factor was 1.01, the correlation (r = 0.056) between ARterm and PT_rc_ was not significant, and the residuals were normally distributed. The PC2FM was expressed with 91 observations and validated on random selections of half of the 182 data observations, i.e. site locations (10). The average tested model coefficient of determination (*r*^*2*^) was 0.78%.

**Fig 1:**
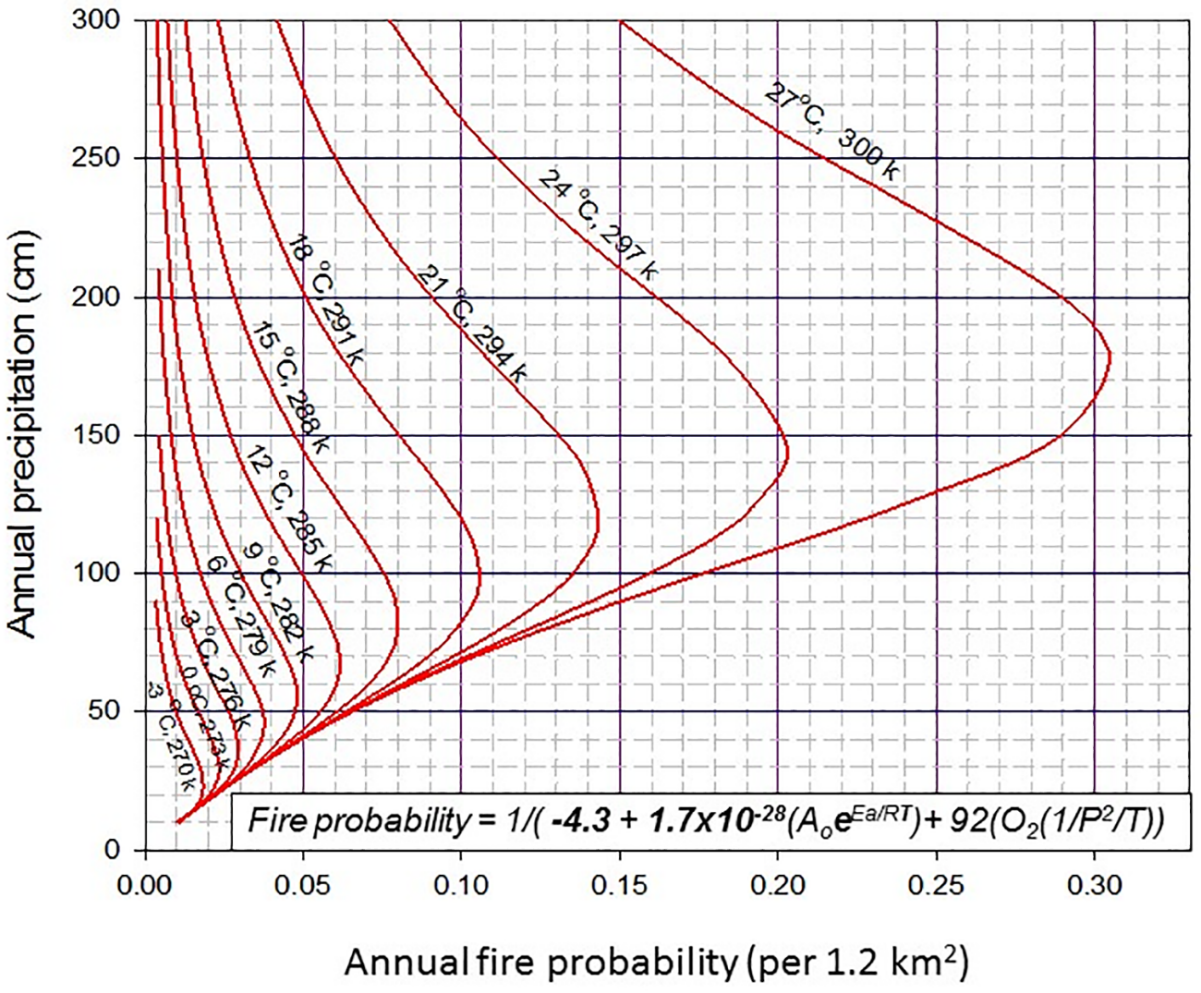
A Combustion-Climate diagram (CCd) of climate influences on fire probability. Climate simulated fire probabilities for ‘natural’ ecosystems using mean maximum temperature and annual precipitation in the PC2FM [10]. This rate diagram explains two temporal differences related to the combustion of ecosystems. Temperature and precipitation affect the reaction rate at the time the reaction occurs while the rate of fuel production determines the fuel concentration and its combustion rate. These two timing conditions differentially determine the rates of the two components of the PC2FM model: ARterm and the PTrc3 (Eq 1). Most ecosystems with adequate carbon bond production fall within the precipitation and temperature limits of this diagram. The red vertical-diagonal isotherms of simulated temperatures are 3°C (3 K) apart. Thus, the y-axis distances between points on the vertical temperature isotherms at the same or different precipitation, estimate fire probability change. For example, with an increase of 30 cm in precipitation (from 135 to 165 cm) and a 3°C in temperature (from 297 to 300 K) results in a change in fire probability from 0.2 to 0.30 or from MFI values of 5 to 3.3 years.

Reaction rates in ecosystem fire are determined by: 1) the conditions of the environment at the time of molecular bond exchanges and 2) the conditions of the fuel production environment (Fig 1). These two processes take ~ seconds (molecular bond exchanges, fire) versus ~ years (fuel production). The importance of this timing is well known to wildland fire managers and fighters but is less well quantified in the fire literature.

## Results and discussion

### Climate spaces as defined by combustion dynamic

**Fig 2:**
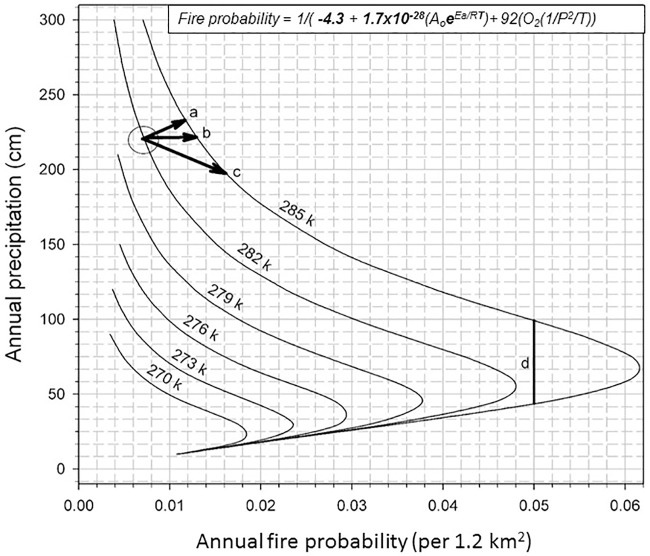
Example of precipitation effects interacting with temperature in a higher resolution CCd diagram of fire probability in cooler (< 285 K) climates. Example of changes in a wet-cool location, the in Lower Elwha River, Olympic Peninsula, Washington [14] where fire probability will be affected by changing temperature and precipitation. Using a 3° change in temperature, from 282 to 285, if annual precipitation increases from 225 to 250 cm (line a) with a 3°C increase in temperature the fire probability with increase to 0.011 (mean interval of 91 years) but less than without precipitation increase. The same temperature increase without a change in precipitation will increase the fire probability from 0.0065 (MFI = 153) to 0.0125 (mean interval = 80 years) (line b). If on the other hand annual precipitation decreases from 225 to 200 cm (line c) with a 3°C increase in temperature the fire probability increases to 0.016 (mean interval of 62 years). The black line (d) illustrates how fire probability can be the same under different annual precipitation along the same temperature isotherm (285 K).

**Fig 3:**
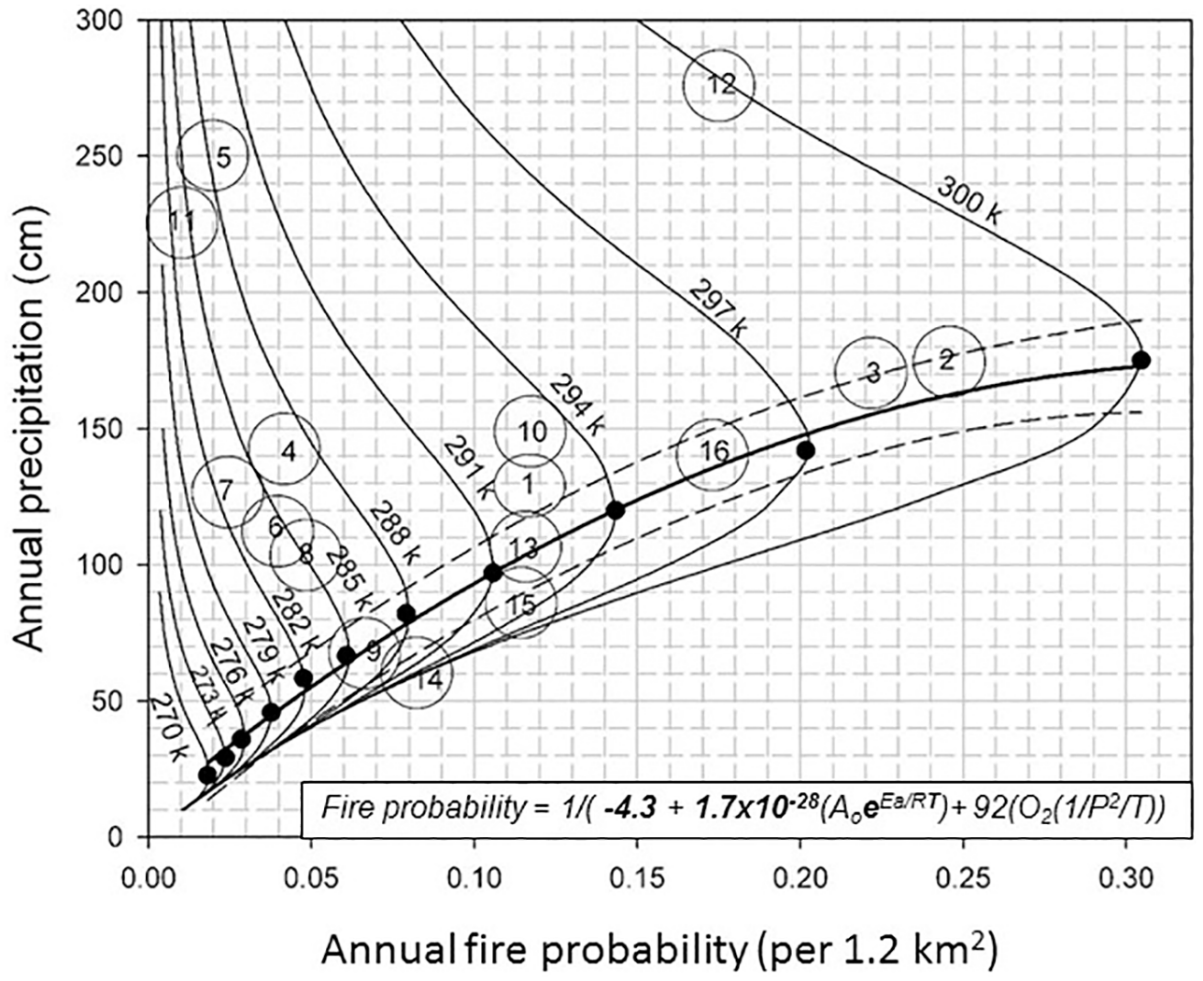
The thick black line (CDL) connecting SWO Loci on each temperature isotherm (solid black dots) separates ecosystems with different fire probability responses to precipitation in the CCd diagram. The CDL (thick black line) separates ecosystems with two dominate precipitation influences on fire probability. Example ecosystems 14 and 15, left of the CDL, may increase in fire probability with more fuel generated by increased plant growth while other wetter ecosystem will decline in fire probability with more humidity and fuel moisture. The SWO Loci (black dots) represent the maximum probability of fire occurrence at a given temperature and precipitation. Circles with numbers are selected ecosystems from Table 2 for illustrative purposes in the diagram’s climate space. These diagramed probability estimates do not match full PC2FM equation predictions, especially at elevations above 2000 m where reduced O_2_ concentrations may become more (or less) significant in Eq 1.

The results, use and theory of this experimental modeling are best expressed in diagrammatic form (Figs 1–3). The results fall into six categories: 1) how a graphic representation of process modeled fire probability, temperature, and precipitation can be used to express fire probability (Fig 1); 2) how the use of physical chemistry and combustion dynamics can produce fire probability estimates that are independent of factors other than climate and rely only on combustion chemistry); 3) how the diagrams can be used to estimate the magnitude and direction of climate forced fire probability changes (Fig 1–3); 4) how the diagrams (Fig 3) classify ecosystems into precipitation insensitive, unstable, and sensitive; 5) how fire regimes in ecosystems are *reaction* (atmospheric conditions) and *reactant* (fuel) *limited*; and 6) how the diagrams do not require the calculation of the PC2FM equation for general user information.

We assess ecosystem fire probability with two factors that are important in all carbon combustion dynamics, the two components of the PC2FM, the ARterm and the PC_rc3_ (Eq 1). Here we define a reaction-limited ecosystem as primarily controlled by the conditions of the reaction environmental such as temperature and precipitation and a reactant-limited ecosystem as controlled primarily by the concentration and quality of the reactants (oxygen and carbon bonds). This primacy in combustion dynamics of precipitation divides the diagram (Fig 3) into reaction-limited (right side) and reactant limited (left side) with reference to the line between combustion dynamic Switch Over Loci (SWO), identified as solid, black circles. However, reaction and reactant processes are always present in all combustion reactions. This dialectic of the chemical process allows for the classing ecosystems into three groups. *Precipitation-sensitive* ecosystems that react rapidly to either increases (> fire probability) or decreases (< fire probability) in precipitation represent one group. *Precipitation-unstable* ecosystems lie directly along the line (CDL) of the SWO Loci (black dots, Fig 3). Small amounts of precipitation radically changes the probability of fire. *Precipitation- insensitive* ecosystems are depicted on the right of the CDL line of SWO and only large (> CDL) changes in precipitation can change fire probability. The maximum fire probability at a given temperature isotherm, the pointed segments of the isotherms on the apex of each isotherm, indicate the location of the SWO Locus of the two effects of precipitation on fire in an ecosystem. Together for all isotherms these SWO Loci indicate a slightly non-near linear separation (the CDL) of fire probability into reaction and reactant limited ecosystems (Fig 3).

### Similar fire probabilities in different climates

The modeling and simulation of combustion dynamics, temperature isotherms, and precipitation conveys a hypothetical question as to whether at a given temperature there can be two states of precipitation interactions that result in equivalent fire probabilities. For example, the 0.05 probability of fire (MFI of 20 years) occurs along the 285 K temperature isotherm at annual precipitation values of 100 cm (reaction limited) and at 43 cm (reactant limited) (Fig 2, line d). These paired fire probability states reflect the counteractive effects of reduced precipitation on fuel and the effects of humidity and fuel moisture on fire probability. Although we have not tested this hypothesis with anything other than modeling results we possess data sets from studies in North America that match in temperature and fire probability, but not in precipitation. These hypothetical fire probability equivalencies may or may not be common but appear to exist among fire scar sites in at least four pairs of actual fire histories (Table 1). Although similar fire frequency probabilities exist in these cases (similar temperatures, different precipitation), the possible fire intensity at the top of the line (d) could be greatly increased by higher fuel loading in more favorable growth climates. Conversely, increased fuel moisture could lessen fire intensity.

**Table 1:** Temperature and fire frequency paired sites with comparable fire probability but different annual precipitation.

Location	Pair	MFI		Pred.prob.	*T* (K)	*P* (cm)	Source
		predicted	actual				
N. Pennsylvania	1w	25	23.8	0.043	286.7	137	[13]
Badlands, S. Dakota	1d	23	17	0.050	286.0	41	[15]
Boston Mts., Arkansas	2w	8.2	16	0.122	293.3	127	[16]
New Mexico & Mexico	2d	7.2	15	0.137	293.3	58	[17]
Talladega Mts., AL	3w	6.4	2.6	0.155	295.7	139	[18]
Santa Catalina Mts., CA	3d	6.6	4.7	0.150	297.1	55	[19]
Grea Lakes	4w	20.9	26	0.048	283.8	84	[20]
SW Montana	4d	20.3	27	0.049	282.7	33	[21]

A *Pair* is denoted by #w for wet and #d for dry. *MFI actual* are site fire scar dated mean fire intervals. *MFI pred*. are PC2FM predicted mean fire intervals. *Pred*. *prob*. are modeled AFrP predicted probabilities (Eq 1). *T* is mean maximum temperature in Kelvin. *P* is annual precipitation in cm. The differences in precipitation (*P)* between the *Pairs* are large (2.2 to 3.8 times) but still have closely matched fire probabilities in predicted and actual frequency metrics (Fig 2, line *d*).

These equal temperature fire probabilities are not the only type of fire probability equivalencies that occur in different climates. Unequal temperature fire probabilities occur when large changes in precipitation co-occur with large temperature changes. This equivalency may seem rare but may be important in future fire probabilities. For example, a three degree (°C) increase in temperature can result in equivalent fire probability when annual precipitation values differ. This usually occurs wholly or in part in precipitation insensitive fire climates to the right of the CDL in Fig 3.

The classification of ecosystems with respect to fire probability and climate interactions (precipitation sensitive, precipitation unstable and precipitation insensitive) is a function of the interaction between precipitation and temperature as expressed, calculated, and calibrated in the AFrP (Eq 1). Using this combustion formulation as graphed (Figs 1–3) illustrates that increases in precipitation will rapidly increase fire probability in dry and warm ecosystems to the left of the line of SWO loci (CDL) but more gradually decrease fire probability in moist ecosystems above the CDL of SWO Loci. The effects of this phenomenon are mapped for the continental US [10].

The steeper slopes of the isotherms associated with lower precipitation values, left side of the CDL (Fig 3) and above line of SWO loci (black dots) indicate more gradual changes in fire probability with respect to precipitation and climate, i.e. reaction limited. On the other hand the lower slope of the isotherms to the right of the SWO Loci line (CDL) suggest more rapid changes in fire probability in the reactant limited and precipitation sensitive ecosystems. Because of this difference in fire drivers, reactant limited ecosystems are more sensitive to small changes in precipitation. Rapid changes in fire probability occur in ecosystems indicated on the left side of the SWO loci line (CDL) more so than in ecosystems to the right with changes in precipitation.

### Climate space and timing for fire probability

The temporal dimensions of the ARterm and the PTrc3 terms in structure of the ecosystem level fire regimes (PC2FM) as described in Fig 1 are often a subject of study. Examples of ecosystems’ fire-time relationships (fire intervals and probabilities) are shown in the CCd diagrams climate space (Figs 1–3). Many of these examples exemplify climates with highly variable differences between reaction rates (~seconds) and fuel production rates (~ years). Since fire and vegetation are both primarily influenced by climate over large scales of time and space we expect that categories of ecosystems, temperature, precipitation and wildland fire are associated (Fig 3). In all CCds the diagramed climate space and actual geographic area are not are matched for many reasons, especially since the y-axis of the probability scale reflects non-linear factors.

Fire probability (AFrP) and mean fire interval (MFI) describe the ecological measures of time and fire occurrence. These are the chemical analog of time and likelihood based rate constants in combustion reactions. This analogy works because the reaction environment and reactant quality (concentration and condition) are dominant factors that affect the likelihood of fire occurrence in the laboratory and landscape. The likelihood a fire will ignite and the probability that it will spread are a function of the ecological and chemical characteristics controlled by climate. This model and application works with ignitions as a random variable for this precise reason.

Although the model that is presented in the Introduction was generated with fire intervals from 182 global sites, we selected 16 sites from that group to examine widespread geographic data to emphasize the point of global climate effects on fire probability. The variance explained (*r*^*2*^) by the predicted fire frequency (annual probability or MFI) of measured fire intervals (*n = 16*) at fire history sites in diverse climates of world is about 0.91 (*p < 0*.*01*). Table 2 provides detailed site information and predictions.

**Table 2:** Descriptive fire data, including predicted and actual fire probability and mean fire intervals for 16 locations used in the *Combustion-Climate diagram* (CCd) (Fig 3). Fire interval data represent pre fire suppression periods.

Number on Fig 3 and geographic location		Annual fire probability		Predicted MFI (years)	Measured MFI (years)	reference
		Predicted	Actual			
1	Brindabella Range, New South Wales, Australia	0.122	0.167–0.08	8.2	6 to 12	[26]
2	Pine Savannas, Florida, USA	0.20	0.31	4.9	3.2	[27]
3	Kisatchie Wilderness, Louisiana, USA	0.45	0.45	2–4	2.2	[28]
4	Long Branch, N. Pennsylvania, USA	0.043	0.04	22–24	25	[13]
5	Cascade Range Oregon, USA	0.012	0.014	85	72	[29]
6	Rahue, Patagonia, Argentina	0.05	0.058	20	17.2	[30]
7	V. Rayado, Patagonia, Argentina	0.02	0.017	50	59	[30]
8	Millinocket, Maine, USA	0.037	0.019	27	51	[31]
9	Huron Mts, Michigan, USA	0.038	0.038	24	26.2	[32]
10	Middle Tennessee, USA	0.14	0.17	8	6	[33]
11	Olympic Peninsula Washington, USA	0.006	0.006	126–150	160	[14]
12	Western Ghats, India	0.158	0.10	6.3	6, 10, 20	[34]
13	Current River, Missouri, USA	0.125	0.125	7.2	8	[35]
14	Chiricahua National Monument, Arizona	0.132	0.131	12.4	7.6	[36]
15	Wichita Mts., Oklahoma USA	0.121	0.122	5.3	8.2	[33]
16	Talladega Mts., Alabama USA	0.155	0.156	2.6	6.4	[18]

Perhaps the most valuable aspect of developing combustion-climate diagrams (CCd) of climate influences on fire probability can be the subsequent questions and understanding of climate and fire probability. There is a need to further explore the mathematical calculations of SWO related to field observations. Additionally, can the SWO Loci represent a quantitative ‘tipping point’ in the physical-chemistry processes of water in the combustion dynamics of ecosystems? Finally, can ecosystem processes be inferred in the slopes of the temperature isotherms on either side of the CDL?

### Model prediction and validation

The originally model’s [10] power and the shape of the precipitation- temperature ‘fields’ are supported by global fire frequency studies and data in Table 2. The basis of site selection was available fire frequency data from diverse global climates [5]. Highly variable world climates show how precipitation and temperature relate to historic rates of fire intervals. Model predicted simulations are correlated (*r* = .87, *n* = 16) with actual MFIs (columns 4 and 5).

## Conclusions

Using precipitation, temperature and oxygen to describe the combustion dynamics of ecosystems not only allows for descriptors of past and future fire regimes, but can be used to address process (fire) deficits in ecosystems [22 – 25]. Ecosystem Combustion-Climate diagrams enable land managers to estimate future fire probabilities from given temperature and precipitation data. Understanding the change in fire probability based on combustion dynamics of atmosphere and fuels can determine fuels programs [24]. Combustion dynamic categories in ecosystems, 1) precipitation insensitive, 2) precipitation unstable and 3) precipitation sensitive, allow long-term management and steady improvement in climate–fire models and understanding. Whether an increase in precipitation results in more or less fire depends strongly on temperature. Although this concept may be expected, it has not been quantified and used predictively. Other applications of this approach include:

1. Defining where the SWO locus (‘climate tipping’) is in a given ecosystem to understand the fire-limiting factors or drivers of fire
2. Understanding how increased precipitation in a precipitation unstable ecosystem increases fire probability.
3. Examining the relevance of fuel (reactant) reduction to adjust fire frequency in a precipitation insensitive ecosystem.

The theory and quantitative results from the physical chemistry of combustion dynamics may be overshadowed by other factors, such as fuel loading and weather. Continued research can examine combustion dynamics in a variety of ecosystems and at a more fine scale resolution, such as the effect of inter and intra-annual variation in temperature and precipitation. The approach described in this research provides another tool for climate scientists, fire scientists, and ecosystem managers.
